# Quadratus lumborum block vs femoral/fascia iliaca block for hip surgeries: A systematic review and meta-analysis

**DOI:** 10.12669/pjms.41.4.11531

**Published:** 2025-04

**Authors:** Yufan Wang, Lina Zhu

**Affiliations:** 1 Yufan Wang, Department of Anesthesiology, Zhejiang Rongjun Hospital, 309 Shuangyuan Road, Jiaxing, Zhejiang Province 314000, P.R. China; 2 Lina Zhu, Department of Anesthesiology, Zhejiang Rongjun Hospital, 309 Shuangyuan Road, Jiaxing, Zhejiang Province 314000, P.R. China

**Keywords:** Analgesic consumption, Fascia iliaca block, Femoral nerve block, Hip arthroplasty, Quadratus lumborum block, Regional anesthesia

## Abstract

**Objective:**

The current systematic review was conducted to compare quadratus lumborum block (QLB) vs fascia iliaca block (FIB) and femoral nerve block (FNB) for improving analgesic outcomes in patients undergoing hip surgeries.

**Methods:**

We searched for randomized controlled trials from inception to on Embase, PubMed, Web of Science, clinical trial registry, and Google Scholar comparing QLB vs FIB/FNB for hip surgeries. The search was initiated on 1^st^ December and culminated on 5^th^ December 2023 to include all studies published from inception till the last day of the search. The primary outcome was 24 hours total analgesic consumption in morphine equivalents. Secondary outcomes were pain scores and incidence of quadriceps weakness at 24 hours, and postoperative nausea and vomiting (PONV).

**Results:**

Six RCTs were eligible. The meta-analysis found that 24-hours morphine consumption was found to be significantly lower in the FIB/FNB group as compared to the QLB group. Pain scores on the 10-point scale were not significantly different between the two groups at one to two hours, two to four hours, 12 hours, 24 hours, and 48 hours. Incidence of quadriceps weakness and PONV was also not significantly difference between the two groups.

**Conclusion:**

Meta-analysis of a limited number of RCTs shows that QLB does not provide better postoperative analgesia as compared to FIB/FNB after hip surgery. Twenty four hours total opioid consumption was significantly higher with QLB but without any difference in pain scores. Incidence of quadriceps weakness and PONV does not differ between QLB and FIB/FNB.

## INTRODUCTION

Postoperative pain control is an essential component of patient management after hip surgeries. Inadequate analgesia can lead to higher complication rates, increased healthcare costs and be a source of patient dissatisfaction.^1^ The importance of pain control cannot be underestimated in hip surgeries since most of the patients are elderly and require optimum analgesia to allow early rehabilitation.^2^ As with all orthopaedic procedures, opioids are the cornerstone of pain management after hip surgeries. However given the high incidence of adverse events of opioids especially in the elderly who have altered hemodynamics and comorbid medical conditions,^3^ there is a need for alternative methods.

Regional anaesthetic modalities like epidural anaesthesia, peripheral nerve blocks and fascial blocks have gained popularity in the past two decades to reduce opioid consumption and improve recovery in various surgical procedures.^4^ For hip surgeries, the fascia iliaca block (FIB), femoral nerve block (FNB), three-in-one block, lumbar plexus block, pericapsular nerve block, quadratus lumborum block (QLB), erector spinae plane block have emerged as alternative regional anaesthetic modalities but with variable results.^5^ȓ^7^ To date, there is no evidence on which modality provides the best analgesia after hip surgery.

Due to ease of access, the most commonly used techniques are the FIB and FNB.^7^ The FNB is an easily administered direct nerve block but with potential complications like femoral nerve injury and damage to femoral vessels, while the FIB is a more proximal block to remotely anaesthetize the femoral nerve without potential injury to the neurovascular bundle.^8^ In the FIB, a large volume of injectate is deposited deep to the fascia iliaca to potentially anaesthetize the femoral, obturator and lateral femoral cutaneous nerves. However, the spread of anesthetic to the obturator nerve has been questioned and FIB may considered only as a proximal FNB.^9^ Meta-analysis studies have shown equivalent efficacy of both FIB and FNB for hip surgeries.^10^,^11^

The QLB is a new regional fascial block which targets the thoracolumbar fascia.^12^ It has become a popular pain control modality for various lower abdominal surgeries.^13^ Based on the needle position around the quadratus lumborum muscle various types of QLB have been described. Specifically, the anterior QLB has the potential to anesthetize the lumbar plexus and hence offer more proximal and wide blockade of the area of interest in hip surgeries.^14^ However, in clinical practice does the QLB demonstrate better results than the FIB/FNB? To answer this query, the current systematic review and meta-analysis was conducted to examine the analgesic efficacy of QLB vs FIB/FNB for hip surgeries.

## METHODS

RCTs were retrieved from literature by two reviewers in collaboration with a medical librarian experienced in systematic reviews. A detailed search of several databases (Embase, PubMed, and Web of Science) was done to identify English language studies comparing QLB and FIB/FNB for hip surgeries. The search was initiated on 1^st^ December and culminated on 5^th^ December 2023 to include all studies published from inception till the last day of the search. We also searched https://clinicaltrials.gov for any completed trials which have posted results but not published. Google Scholar was scanned for any missed studies.

### Registration

The study was based on the PRIMA guidelines.^15^ The protocol of the review has been registered on PROSPERO and can be accessed at https://www.crd.york.ac.uk/prospero/ with the registration number, CRD42023476727.

The search strategy is available as Supplementary Table-I and includes a combination of the keywords: “Quadratus lumborum block; Fascia iliaca block; Femoral nerve block; Hip surgery; Hip arthroplasty; and Femoral neck”. The search strategy combined these keywords into various combinations with “AND” and “OR” to optimize the results.

**Supplementary Table-I:** Details of the search queries.

Query	Search Details
(quadratus lumborum block) AND (hip arthroplasty)	"quadratus"[All Fields] AND "lumborum"[All Fields] AND ("block"[All Fields] OR "blocked"[All Fields] OR "blocking"[All Fields] OR "blockings"[All Fields] OR "blocks"[All Fields]) AND (("hip"[MeSH Terms] OR "hip"[All Fields]) AND ("arthroplasty"[MeSH Terms] OR "arthroplasty"[All Fields] OR "arthroplasties"[All Fields]))
(quadratus lumborum block) AND (hip surgery)	"quadratus"[All Fields] AND "lumborum"[All Fields] AND ("block"[All Fields] OR "blocked"[All Fields] OR "blocking"[All Fields] OR "blockings"[All Fields] OR "blocks"[All Fields]) AND (("hip"[MeSH Terms] OR "hip"[All Fields]) AND ("surgery"[MeSH Subheading] OR "surgery"[All Fields] OR "surgical procedures, operative"[MeSH Terms] OR ("surgical"[All Fields] AND "procedures"[All Fields] AND "operative"[All Fields]) OR "operative surgical procedures"[All Fields] OR "general surgery"[MeSH Terms] OR ("general"[All Fields] AND "surgery"[All Fields]) OR "general surgery"[All Fields] OR "surgery s"[All Fields] OR "surgerys"[All Fields] OR "surgeries"[All Fields]))
(((Femoral nerve block) OR (fascia iliaca block)) AND (quadratus lumborum block)) AND (femoral neck)	((("femoral nerve"[MeSH Terms] OR ("femoral"[All Fields] AND "nerve"[All Fields]) OR "femoral nerve"[All Fields]) AND ("block"[All Fields] OR "blocked"[All Fields] OR "blocking"[All Fields] OR "blockings"[All Fields] OR "blocks"[All Fields])) OR (("fascia"[MeSH Terms] OR "fascia"[All Fields] OR "fasciae"[All Fields] OR "fascias"[All Fields]) AND "iliaca"[All Fields] AND ("block"[All Fields] OR "blocked"[All Fields] OR "blocking"[All Fields] OR "blockings"[All Fields] OR "blocks"[All Fields]))) AND ("quadratus"[All Fields] AND "lumborum"[All Fields] AND ("block"[All Fields] OR "blocked"[All Fields] OR "blocking"[All Fields] OR "blockings"[All Fields] OR "blocks"[All Fields])) AND ("femur neck"[MeSH Terms] OR ("femur"[All Fields] AND "neck"[All Fields]) OR "femur neck"[All Fields] OR ("femoral"[All Fields] AND "neck"[All Fields]) OR "femoral neck"[All Fields])
(((Femoral nerve block) OR (fascia iliaca block)) AND (quadratus lumborum block)) AND (hip surgery)	((("femoral nerve"[MeSH Terms] OR ("femoral"[All Fields] AND "nerve"[All Fields]) OR "femoral nerve"[All Fields]) AND ("block"[All Fields] OR "blocked"[All Fields] OR "blocking"[All Fields] OR "blockings"[All Fields] OR "blocks"[All Fields])) OR (("fascia"[MeSH Terms] OR "fascia"[All Fields] OR "fasciae"[All Fields] OR "fascias"[All Fields]) AND "iliaca"[All Fields] AND ("block"[All Fields] OR "blocked"[All Fields] OR "blocking"[All Fields] OR "blockings"[All Fields] OR "blocks"[All Fields]))) AND ("quadratus"[All Fields] AND "lumborum"[All Fields] AND ("block"[All Fields] OR "blocked"[All Fields] OR "blocking"[All Fields] OR "blockings"[All Fields] OR "blocks"[All Fields])) AND (("hip"[MeSH Terms] OR "hip"[All Fields]) AND ("surgery"[MeSH Subheading] OR "surgery"[All Fields] OR "surgical procedures, operative"[MeSH Terms] OR ("surgical"[All Fields] AND "procedures"[All Fields] AND "operative"[All Fields]) OR "operative surgical procedures"[All Fields] OR "general surgery"[MeSH Terms] OR ("general"[All Fields] AND "surgery"[All Fields]) OR "general surgery"[All Fields] OR "surgery s"[All Fields] OR "surgerys"[All Fields] OR "surgeries"[All Fields]))

### Eligibility criteria:

- PICOS inclusion criteria was formulated for the review. It was as follows:
- Population-Adult patients undergoing any major open hip surgery like hip arthroplasty or surgery for hip fracture
- Intervention-Any type of QLB given in the perioperative period
- Comparison-FNB or supra/infra-inguinal FIB
- Outcomes-24 hour total opioid consumption or pain scores
- Study type-Randomized controlled trials (RCTs)

All studies on hip arthroscopy, non-RCTs, and studies not reporting relevant outcomes were excluded.

### Study selection:

The results of the three databases (Embase, PubMed, and Web of Science) were then combined in the reference manager software. Any trials found by additional searching were then added. All duplicate studies were eliminated and the rest were scanned based on the inclusion criteria. The reviewers identified relevant studies and read their full texts. Studies fulfilling all criteria were included. The references of these studies were also searched for any missed trials. Any disagreements between reviewers were resolved by discussion.

### Extracted data and outcomes:

A data extraction form was used to collect all data from the studies by two reviewers. Any variations were then cross-checked and corrected by discussion. The author’s name, year of publication, country, type of hip surgery, method of QLB, method of FNB/FIB, sample size, age and gender details, patient position, anaesthetic used, type of rescue analgesic, any other analgesics used in the postoperative period, and outcomes. The primary outcome was 24-hour total opioid consumption in morphine equivalents. Secondary outcomes were pain scores at one to two hours, four to six hours, 12 hours, 24 hours and 48 hours, incidence of muscle weakness at 24 hours, and postoperative nausea and vomiting (PONV). If data was incompletely reported, the corresponding author was contacted once on email.

### Evaluation of study quality:

Study quality was examined in all RCTs by two reviewers using the Cochrane Collaboration risk of bias-2 tool.^16^ The RCTs were assessed for random sequence generation and allocation concealment, deviation from intended intervention, missing data, measurement of outcomes by blinding, selection of reported results, and overall risk of bias. Each item was rated as “high risk”, “low risk, or “some concerns”.

### Statistical analysis:

Pooled analysis of data was performed using “Review Manager” (RevMan, version 5.3; Nordic Cochrane Centre (Cochrane Collaboration), Copenhagen, Denmark; 2014). As all studies had methodological heterogeneity we preferred the random-effects meta-analysis model. Continuous and binary outcomes were pooled to generate mean difference (MD) and odds ratio (OR) respectively with 95% confidence intervals (CI). If continuous data was reported as a median and interquartile range, data was converted using the methods of Wan et al.^17^ Engauge digitizer was used for obtaining data from graphs.

Due to a low number of studies funnel plots were not generated. The I^2^ statistic was the tool to determine inter-study heterogeneity. I^2^ <50% meant low and >50% meant substantial heterogeneity. Sensitivity analysis was done for the primary outcome to check the robustness of results by eliminating one study at a time. P-values below 0.05 were considered significant.

## RESULTS

**Fig.1:**
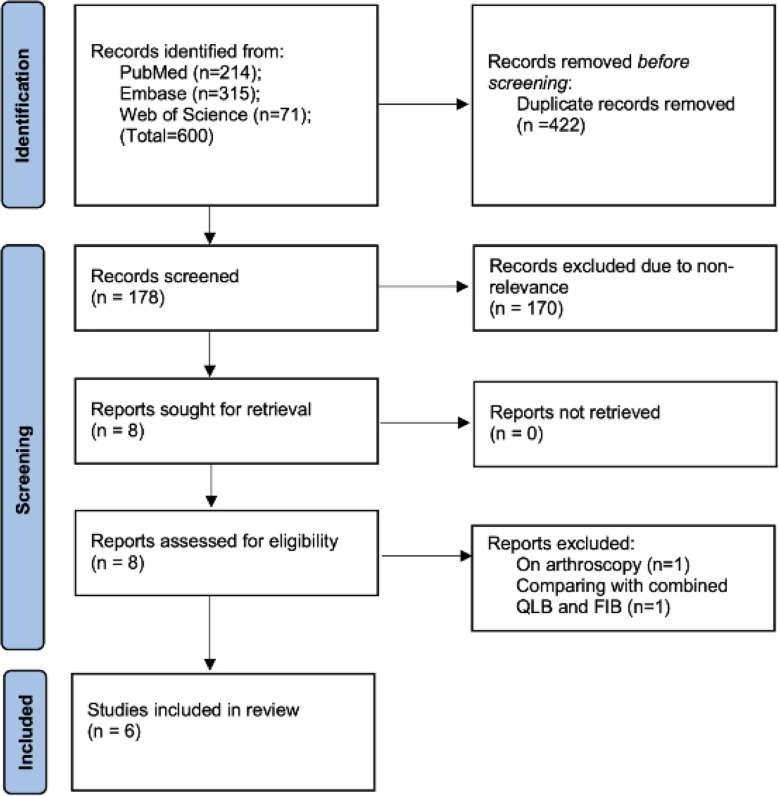
PRISMA flow-chart showing study selection.

The pooled total number of search results for the three databases was 600. No new studies were retrieved on https://clinicaltrials.gov and Google Scholar. After deduplication, 178 articles were screened and eight were found to be relevant to the review. The inter-reviewer agreement was found to be high (kappa=0.95). Six RCTs^18^ȓ^23^ were finally included (Fig.1). Details are shown in Table-I. These studies were published between the years 2020 to 2023. Except for one study on hip fracture, all other studies were on total hip arthroplasty. Three used general anesthesia while three used spinal anesthesia. All blocks were given preoperatively and under ultrasound guidance. All studies used the anterior QLB (also known as the transmuscular QLB). QLB was compared with FNB in two studies and FIB in the remaining studies. One study used continuous blocks in both groups. The dose, volume and concentration of the anaesthetic were variable across studies. The local anaesthetics used were bupivacaine, levobupivacaine, and ropivacaine. Post-operative analgesic protocol and type of rescue analgesia were also quite variable among the studies. Supplementary Table-II shows the reviewer’s judgement on the risk of bias. Four studies were high quality with a low risk of bias. Two RCTs had high risk of bias.

**Table-I:** Baseline details of included studies.

Study	Location	Surgery	QLB protocol	Control group protocol	Groups	Sample size	Mean age (years)	Male gender (%)	Rescue analgesic	Other analgesics
Takeda 2023	Japan	Total hip arthroplasty under GA	Preoperative USG guided anterior QLB performed in lateral position with 20 ml of 0.5% levobupivacaine and 10ml of saline	Preoperative USG guided FNB performed in supine position with 10 ml of 0.5% levobupivacaine and 5ml of saline	QLB	54	69	4.3	PCA with fentanyl. Delivering 3mL/dose (0.6 mg/kg) of fentanyl on demand with a 30 min lockout interval and no background infusion	IV PCM twice every 6 h on POD-1
					Control	56	71	30		
Refaat 2023	Egypt	Hip surgery for femoral neck fracture under SA	Preoperative USG guided anterior QLB performed in lateral position with 40 ml of 0.25% bupivacaine	Preoperative USG guided suprainguinal FIB performed in supine position with 40 ml of 0.25% bupivacaine	QLB	34	53.4	50	IV Morphine 5mg	IV PCM 15mg/kg every 6 h on POD-1
					Control	34	55.5	55.9		
Wang 2022	China	Total hip arthroplasty under GA	Preoperative USG guided anterior QLB performed in lateral position with 30 ml of ropivacaine 0.33% with epinephrine 2 mg/mL	Preoperative USG guided suprainguinal FIB performed in supine position with 30 ml of ropivacaine 0.33% with epinephrine 2 mg/mL	QLB	50	53.2	54	SC morphine 10mg	Oral celecoxib 200mg twice daily
					Control	50	54.7	58		
Hashmi 2022	Ireland	Total hip arthroplasty under SA	Preoperative USG guided transmuscular QLB performed in lateral position with 20 ml of 0.25% bupivacaine	Preoperative USG guided infrainguinal FIB performed in supine position with 20 ml of 0.25% bupivacaine	QLB	24	NR	39.3	IV morphine in 2mg increments every 5 min up to 10mg in PACU. PCA morphine at 1 mg/mL concentration, 1 mg dose, 5 min lockout period, and max 40 mg in 4 h for 24 h	Oral PCM 1g every 6 h and oral celecoxib 200 mg every 12 h
					Control	24		60.7		
Nassar 2021	Egypt	Total hip arthroplasty under SA	Preoperative USG guided transmuscular QLB performed in lateral position with 30 ml of 0.25% bupivacaine	Preoperative USG guided infrainguinal FIB performed in supine position with 30 ml of 0.25% bupivacaine	QLB	17	54	40	IV morphine in 2mg increments up to 4 mg	Oral PCM 1g every 6 h and oral ketorolac 30mg every 12 h
					Control	19	47	60		
Aoyama 2020	Japan	Total hip arthroplasty under GA	Preoperative USG guided anterior QLB performed in lateral position with 30 ml of 0.25% levobupivacaine followed by insertion of a catheter. Postoperative continuous infusion of 0.125% levobupivacaine at 4 ml/h via the catheter.	Preoperative USG guided FNB performed in supine position with 15 ml of 0.5% levobupivacaine followed by insertion of a catheter. Postoperative continuous infusion of 0.125% levobupivacaine at 4 ml/h via the catheter.	QLB Control	13 11	70 67	15.4 27.3	PCA of QLB/FNB, 3ml per press with lockout of 30 min. IV PCM or diclofenac suppository.	Oral celecoxib 200mg/day.

QLB, quadratus lumborum block; FIB, fascia iliaca block; FNB, femoral nerve block; GA, general anesthesia; SA, spinal anesthesia; USG, ultrasonography; PCA, patient controlled analgesia; POD, postoperative day; PCM, paracetamol; IV, intravenous; SC, subcutaneous; PACU, post-anesthesia care unit

**Supplementary Table-II:** Risk of bias analysis.

Study	Randomization process	Deviation from intended intervention	Missing outcome data	Measurement of outcomes	Selection of reported result	Overall risk of bias
Takeda 2023	Low risk	Low risk	Low risk	Low risk	Low risk	Low risk
Refaat 2023	Some concerns	Low risk	Low risk	High risk	Low risk	High risk
Wang 2022	Low risk	Low risk	Low risk	Low risk	Low risk	Low risk
Hashmi 2022	Low risk	Low risk	Low risk	Low risk	Low risk	Low risk
Nassar 2021	Low risk	Low risk	Low risk	Low risk	Low risk	Low risk
Aoyama 2020	Some concerns	Low risk	Low risk	High risk	Low risk	High risk

**Fig.2:**
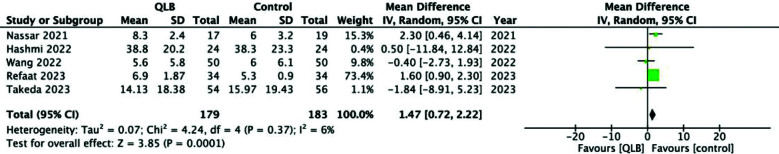
Forest plot comparing 24-hours total opioid consumption between QLB and FIB/FNB in patients undergoing hip surgery.

The meta-analysis found that morphine consumption was significantly lower in the FIB/FNB group as compared to the QLB group (MD: 1.47 95% CI: 0.72, 2.22). Inter-study heterogeneity was low (I^2^=6%) (Fig.2). During sensitivity analysis, the exclusion of Refaat et al,^23^ turned the results non-significant (MD: 0.92 95% CI: -0.95, 2.79 I^2^=23%).

**Supplementary Fig.1:**
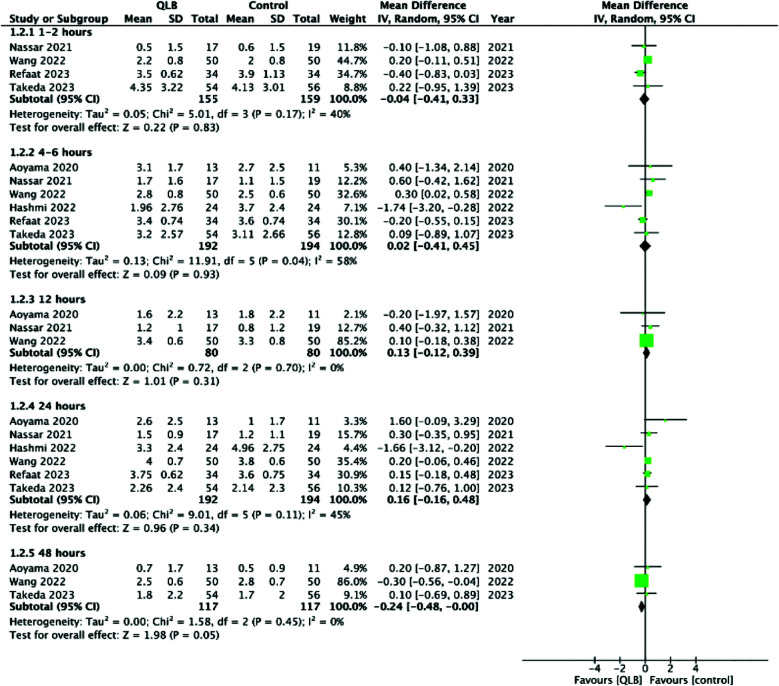
Forest plot comparing pain scores at different times between QLB and FIB/FNB in patients undergoing hip surgery.

**Supplementary Fig.2:**
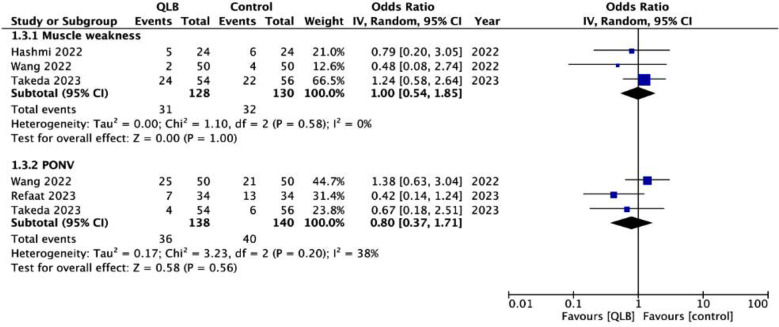
Forest plot comparing the incidence of muscle weakness and PONV between QLB and FIB/FNB in patients undergoing hip surgery.

Pain scores on the 10-point scale did not differ between the two groups at one to two hours (MD: -0.04 95% CI: -0.41, 0.33 I^2^=40%), two to four hours (MD: 0.02 95% CI: -0.41, 0.45 I^2^=58%), 12 hours (MD: 0.13 95% CI: -0.12, 0.39 I^2^=0%), 24 hours (MD: 0.16 95% CI: -0.16, 0.48 I^2^=45%), and 48 hours (MD: -0.24 95% CI: -0.48, -0.00 I^2^=0%) (Supplementary Fig.1). Incidence of muscle weakness (OR: 1.00 95%CI: 0.54, 1.85 I^2^=0%) and PONV (OR: 0.80 95%CI: 0.37, 1.71 I^2^=38%) was also not significantly different between the two groups (Supplementary Fig.2).

## DISCUSSION

Our review is the first meta-analysis comparing the QLB and the FIB/FNB for patients undergoing major hip surgeries. We found that 24 hours total opioid consumption was higher with QLB as compared to FIB/FNB. Out of the five studies in the meta-analysis, three reported no difference in morphine consumption between the two groups while the study of Refaat et al.^23^ and Nassar et al^19^ found higher morphine consumption in the QLB group. In terms of pain scores, our review showed no difference between the QLB and the FIB/FNB groups at any time point. Thus, the outcomes of our study show that QLB is not superior to the traditional regional anaesthetic modalities of FIB and FNB for patients undergoing major hip surgeries.

The productivity of QLB for patients undergoing hip surgery has also been questioned by other reviews. Hussain et al. assessed the benefits of adding the QLB to general or spinal anaesthesia in patients undergoing hip surgeries.^24^ They found that pain scores at rest were improved by the addition of QLB to both general and spinal anaesthesia but the difference failed to reach the minimal clinically important difference of 1.86 on a 10-point scale. The authors concluded that while QLB produced a statistically significant reduction in pain scores, the results may be unimportant for patients and hence do not recommend the use of QLB. Contrastingly, a recent meta-analysis by Hu et al showed that QLB produced a significant and clinically important reduction of pain scores on mobilization as compared to no block.^25^ QLB also improved patient satisfaction scores and led to a reduction in PONV. However, it failed to demonstrate any clinically important difference in pain scores at rest and opioid consumption.

The QLB was first reported by Blanco et al.^12^ Four approaches to this block have been described, namely, anterior, lateral, posterior, and intramuscular.^26^ In all of the studies in this review, the anterior QLB was used wherein the anaesthetic was injected between the psoas major and quadratus lumborum muscle. As the nerves of the lumbar plexus run between these muscles, the anterior QLB has been postulated to provide anaesthesia to the lower extremities as well. A more proximal blockade of the femoral nerve at the level of the lumbar plexus itself can therefore provide anesthesia to a wider operative field.^14^ Nevertheless, questions have been raised on the ability of anterior QLB to reach the lumbar roots with variable results of cadaveric studies.

A cadaveric study by Carline et al has shown that the anterior QLB consistently spread to the lumbar roots L1 to L3.^27^ Another one by Adhikary et al involving only five cadavers showed that the injectate in the anterior QLB stained the upper branches of lumbar plexus and psoas major muscle in 70% of the specimens.^28^ A larger study by Dam et al demonstrated that after the anterior QLB dye spread is predominantly towards the thoracic paravertebral and intercostal space with caudal spread to the subcostal, iliohypogastric, and ilioinguinal nerves in all cases.^29^ In none of the cases, dye was seen to surround the lumbar sympathetic trunk, lumbar plexus, or the femoral nerve. These wide variations in the spread of injectate explain the variable efficacy of QLB reported in different reviews for hip surgeries. It also highlights that anatomical and technical variations in the performance of the block could be important factors affecting the extent of anaesthesia. Indeed, the FIB and FNB are technically more straightforward blocks. The QLB, on the other hand, is a deeper block requiring skill to appropriately direct the needle in the correct plane and may require hydrodissection of the space before local anaesthetic injection.^20^

Studies have shown that QLB does not affect postoperative ambulation as compared to no block in patients undergoing hip surgeries.^30^,^31^ In our review, we found no difference in the risk of muscle weakness between QLB and FIB/FNB groups. Similarly, the risk of PONV was also not different between the two groups. The outcomes must be interpreted with caution since only three studies were available for data analysis. Interpretation of muscle weakness is also dependent on the examiner. Only future data from large RCTs can provide robust evidence on motor weakness.

### Strengths

The strengths of the review include a comprehensive and systematic literature search and a detailed meta-analysis to present for the first time, high-quality evidence on the comparative efficacy of QLB vs standard regional anesthetic modalities of FIB and FNB for hip surgeries. The review also has important clinical implications. The current results do not encourage the use of QLB instead of FIB or FNB in hip surgery patients. The easier-to-perform FIB and FNB may provide better analgesia and reduce opioid requirements in hip surgery patients.

### Limitations

This review has several potential limitations. The number of RCTs available for analysis was not high. Secondly, inter-study methodological heterogeneity is an important limitation. Variations in the dose, volume, type of anaesthetic, type of rescue analgesic, and postoperative analgesic protocol could have impacted results. The experience of clinicians performing the block is also an important confounder which is difficult to control. Thirdly, due to limited studies, a subgroup analysis of FIB and FNB was not possible. Lastly, out of the five studies, two RCTs had a high risk of bias primarily due to lack of blinding.

Given the limitations, we recommend that further robust RCTs be conducted to assess the relevance of QLB for hip surgery. Such studies should control for anesthetic dose, volume, type of anaesthetic, type of rescue analgesic, and postoperative analgesic protocol to obtain high-quality evidence. Studies should also conduct a three-way comparison of QLB, FIB and FNB to better segregate evidence.

## CONCLUSIONS

Meta-analysis of a limited number of RCTs shows that QLB does not provide better postoperative analgesia as compared to FIB/FNB after hip surgery. Twenty four hours total opioid consumption was slightly but significantly higher with QLB but without any difference in pain scores. Further robust RCTs can aid in improving current evidence.

### Authors’ contributions:

**YW:** Study design, literature search and manuscript writing.

**YW and LZ:** Data collection, data analysis and interpretation, critical review.

**YW:** Was involved in the manuscript revision and validation.

All authors have read, approved the final manuscript and are accountable for the integrity of the study.
